# The Cultural Dynamics of Copycat Suicide

**DOI:** 10.1371/journal.pone.0007252

**Published:** 2009-09-30

**Authors:** Alex Mesoudi

**Affiliations:** Biological and Experimental Psychology Group, School of Biological and Chemical Sciences, Queen Mary, University of London, London, United Kingdom; Stanford University, United States of America

## Abstract

The observation that suicides sometimes cluster in space and/or time has led to suggestions that these clusters are caused by the social learning of suicide-related behaviours, or “copycat suicides”. *Point clusters* are clusters of suicides localised in both time and space, and have been attributed to direct social learning from nearby individuals. *Mass clusters* are clusters of suicides localised in time but not space, and have been attributed to the dissemination of information concerning celebrity suicides via the mass media. Here, agent-based simulations, in combination with scan statistic methods for detecting clusters of rare events, were used to clarify the social learning processes underlying point and mass clusters. It was found that social learning between neighbouring agents did generate point clusters as predicted, although this effect was partially mimicked by homophily (individuals preferentially assorting with similar others). The one-to-many transmission dynamics characterised by the mass media were shown to generate mass clusters, but only where social learning was weak, perhaps due to prestige bias (only copying prestigious celebrities) and similarity bias (only copying similar models) acting to reduce the subset of available models. These findings can help to clarify and formalise existing hypotheses and to guide future empirical work relating to real-life copycat suicides.

## Introduction

While suicide is undoubtedly a complex phenomenon with multiple and diverse causes [1], [2], evidence accumulated over recent years suggests that one of these causes may be *social learning*. These “copycat” suicides are proposed to be caused at least in part by exposure to another individual's suicide, for example through the imitation of suicidal behaviour. Two general patterns of suicide clusters have been documented and taken as evidence for copycat suicides [3]: point clusters, which are localised in both time and space, and mass clusters, which are localised in time only.

A *point cluster* is defined as a temporary increase in the frequency of suicides within a small community or institution, relative to both the baseline suicide rate before and after the point cluster and the suicide rate in neighbouring areas [4], [5]. For example, Haw [6] documented 14 suicides within a psychiatric hospital during a one-year period, while Brent et al. [7] documented two suicides and seven suicide attempts during a 14-day period in a single school. Beyond anecdotal case studies, Gould et al. [5] used statistical analyses designed to detect the clustering of disease infections to determine whether suicides occur in spatiotemporal clusters. On average around 2% of suicides amongst 15–19 year olds in the U.S. were found to cluster spatially and temporally beyond that expected by chance, although this figure was as high as 13% in some states. Given this spatiotemporal clustering, point clusters are frequently explained in terms of copycat suicides, with suicidal behaviour spreading through a local network via social learning [4], [5].

A *mass cluster* is defined as a temporary increase in the total frequency of suicides within an entire population relative to the period immediately before and after the cluster, with no spatial clustering. Mass clusters are typically associated with high-profile celebrity suicides that are publicised and disseminated in the mass media. Analyses have shown that national suicide rates rise immediately after the suicides of entertainment celebrities, and to a lesser extent political figures, have been highly publicised in the mass media [8]–[10]. The implication here is that this rise is caused by social learning: people across the country imitate the suicide behaviour of the celebrity. Consistent with a social learning effect, this increase is found to be proportional to the amount of media coverage, e.g. the number of column inches devoted to the suicide [8] or the number of television networks covering the suicide [10]. Moreover, suicide rates do not show a corresponding drop some time after the publicised suicide, suggesting that the immediate increase is not caused by already-vulnerable people committing suicide earlier than they otherwise would have [8]. The effect appears to be restricted to the suicides of famous people who are afforded some degree of prestige in their society (e.g. entertainment celebrities); in contrast, non-celebrities and famous figures who have negative reputations (e.g. cold war spies), both of whom lack prestige, have smaller or non-significant effects on national suicide rates [9], [10]. There is also evidence that people are more likely to imitate the suicides of celebrities who match them in gender and nationality [9], although this effect is less robust than the celebrity effect [11]. Similar increases in suicide rates in response to media-publicised suicides have been observed in Germany [12], Japan [13], Taiwan [14] and Austria [15].

The overall aim of this study is to use agent-based simulations to formally explore how different social learning dynamics might generate these different spatial and temporal clusters of suicides. Agent-based models are typically used in cases such as these to determine the population-level patterns generated by underlying interactions between individuals [16], [17], making them a useful tool in the case of copycat suicides. Specifically, the agent-based model addresses possible explanations that have been posited for each of the two kinds of suicide clusters - point and mass - as discussed below.

### Point clusters: Social learning or homophily?

Joiner [3] has challenged the assumption made by researchers such as Gould et al. [5] that spatiotemporal point clusters are necessarily caused by social learning. Joiner [3] hypothesised that point clusters may instead be a by-product of *homophily*, the tendency for similar individuals to preferentially associate with one another [18]. If people preferentially associate on the basis of factors that increase the risk of (non-copycat) suicide, then spatial clusters of high-risk people will emerge. These high-risk clusters may form suicide clusters due to each member's independently high risk of suicide, without any social learning occurring within the cluster. Joiner [3] suggests that many spatiotemporal suicide clusters observed in hospitals and schools may be cases of independent suicides within homophilous groups of high-risk individuals. However, while there is extensive evidence for the general phenomenon of homophily [18], no direct empirical test of Joiner's hypothesis has yet been conducted in relation to suicide, and without such tests it is difficult to determine which of these explanations - copycat suicide via social learning or independent suicide within a homophilous network - is responsible for point suicide clusters.

The first aim of the present study is to determine whether homophily can mimic social learning in generating point clusters, and if so to guide future empirical research by exploring the conditions under which this is most likely to occur.

### Mass clusters: Prestige bias, similarity bias, and/or the mass media?

Explanations for mass suicide clusters have centred around three characteristics of such clusters: (i) that they are associated with prestigious celebrities only, (ii) that the effect is greater when the celebrities are similar to the target individual, and (iii) that the mass media is involved in the dissemination of suicide information. Regarding the first two of these, Henrich and McElreath [19] suggest that mass suicide clusters result from two social learning biases: *prestige bias*, where individuals preferentially copy the behaviour of prestigious or high-status models [20], and *similarity bias*, where individuals preferentially copy the behaviour of models who are similar to them in ethnic markers such as dialect, language or dress [21]. Evolutionary models suggest that both prestige bias and similarity bias are adaptive means of acquiring accurate information compared to both costly trial-and-error individual learning and the unbiased copying of other randomly-chosen people [22]. Prestigious individuals have usually acquired high prestige because their behaviour is adaptive, and so copying prestigious individuals will, on average, lead to the acquisition of that adaptive behaviour [20]. Copying similar individuals is likely to lead to the acquisition of adaptive behaviour because similar individuals face similar adaptive challenges and so should have appropriate solutions to such challenges [21]. Crucially, however, both prestige and similarity bias are vulnerable to the occasional acquisition of maladaptive behaviour when such behaviour is exhibited by prestigious or similar individuals. Thus copycat suicide can be seen as a maladaptive by-product of these generally adaptive social learning rules [19].

Other researchers frequently cite the mass media as a driver of mass suicide clusters [10], [11], [23], with suicide-related behaviour assumed to be disseminated via newspapers, magazines, television and radio. Indeed, this assumption has led to the establishment of guidelines and safeguards concerning the reporting of suicides in the media [11], [23]. Formally, mass media dissemination resembles “one-to-many” cultural transmission [24], where a single individual can influence a large number of other individuals simultaneously. Cultural evolution models suggest that the extreme one-to-many transmission that is permitted by the mass media can greatly increase the rate at which behavioural traits spread [24], thus potentially generating temporal clusters. Note that prestige/similarity bias and one-to-many transmission are not mutually exclusive hypotheses: the “one” individual from whom the “many” learn may be more prestigious than, or similar to, the “many”.

The second aim of the present study is to explore which of the aforementioned social learning biases - prestige bias, similarity bias and/or one-to-many transmission - are necessary and sufficient to generate mass suicide clusters.

### Hypotheses

Based on the literature reviewed above, the following predictions are made:

1a. Social learning generates spatiotemporal point suicide clusters.
1b. Homophily can generate spatiotemporal point clusters in the absence of social learning.
2a. Prestige bias generates temporal (but not spatial) mass suicide clusters.
2b. Similarity bias generates temporal (but not spatial) mass suicide clusters.
2c. One-to-many cultural transmission, i.e. the cultural dynamics characterised by the mass media, generates temporal (but not spatial) mass suicide clusters.

## Methods

The agent-based model was programmed in Borland C++ Builder. An executable (.exe) version is available for download as Supplementary File S1 and may be used to recreate the results presented below. Source code is available upon request from the author.

The freely-available program SaTScan™ [25] was used to detect spatial, temporal, and spatiotemporal clusters in the suicide frequency data generated by the agent-based model. This program is commonly used to detect clustering of diseases in space and time, such as leprosy [26], West Nile virus [27] and gonorrhoea [28]. Previous simulation studies have found that SaTScan™ is effective in detecting clusters of rare events [29] making it particularly applicable to suicides. SaTScan™ uses the scan statistic [30] to identify statistically significant clusters, i.e. clusters that deviate from frequencies expected under a random distribution. A window of varying size is gradually moved across time and/or space and the number of observed events (here, suicides) is compared with the number expected under a random, no-clustering distribution. This window is either an interval in time (for temporal scanning), a circle (for spatial scanning) or a cylinder with a circular spatial base and a time interval as its length (for spatio-temporal scanning). The maximum cluster size was set here at 50% of the total area for spatial clusters and 50% of generations for temporal clusters to avoid biasing the detection with *a priori* target cluster sizes. For each location and/or size of the window, the expected frequency under the null hypothesis of no clustering is calculated assuming a Bernoulli distribution, and the window with the maximum likelihood is identified. The statistical significance of this window is calculated using a Monte Carlo simulation method. The scanning procedure and maximum likelihood test is repeated for 999 randomly generated replications of the data generated under the null hypothesis. The statistical significance (p value) is given by the rank of the maximum likelihood calculated from the real data compared with all ranked maximum likelihoods from the simulated data sets; if the real maximum likelihood falls within the top α proportion of ranked simulated maximum likelihoods, then the null is rejected (e.g. if α = 0.05, the real maximum likelihood must be within the largest 5% of simulated maximum likelihoods to be assigned statistical significance).

To further increase the robustness of the analysis in the present study, data from ten independent runs of the agent-based model for each set of parameter values were analysed using SaTScan™. The results below are given as the proportion (X) of these ten runs that yielded significant clusters at the p<0.005 level (given ten tests, α is Bonferroni corrected to 0.05/10 = 0.005), either in space (X_s_), time (X_t_), or both time and space (X_st_). Thus where X_s_ = 0, X_t_ = 0 or X_st_ = 0 then there is no spatial/temporal/spatiotemporal clustering beyond that expected due to chance, and where X_s_ = 1, X_t_ = 1 or X_st_ = 1 then it is statistically most likely that at least one spatial/temporal/spatiotemporal cluster is present in the data generated by the model.

## Results

### Basic model assumptions

The model assumes N = 1000 agents inhabiting a two-dimensional 10×10 grid, with 100 groups each located at a different Cartesian coordinate and 10 agents in each group. This organisation was intended to simulate the kind of social structure often examined in suicide cluster studies (e.g. [5]) and as such may be abstracted to different levels of social organisation, e.g. a collection of schools/hospitals within a town, towns within a state, or states within a country. The population then undergoes T = 100 generations. During each generation, every agent is cycled through in a random order and commits suicide with a probability that is determined by various parameters described in the following sections and summarised in Table 1. If an agent commits suicide, it is replaced with a new agent and, in the social learning conditions, affects the surrounding agents' probabilities of suicide in the following generation. Each suicide is recorded as a case and the entire 100-generation dataset is analysed for clusters using SaTScan™.

**Table 1:** Definitions of parameters manipulated in the model.

Parameter	Definition
*p_0_*	Baseline (non-copycat) suicide rate
*s*	Social influence, i.e. the increase in *p_0_* in response to another agent committing suicide
*q*	The magnitude of individual differences in suicide risk factors
*h*	Homophily, i.e. the extent to which agents in the same group share risk factors
*c_p_*	The probability that an agent is a prestigious “celebrity”
*c_s_*	Prestige bias, i.e. the increase in *p_0_* in response to the suicide of a prestigious “celebrity” (replaces *s*)
*m*	Similarity bias, i.e. the number of binary risk factors (out of six) that a model and an observer must share in order for the former to exert social influence on the latter
*r*	One-to-many transmission, i.e. the radius of the circular area around a suicide agent across which social influence is exerted

During the analysis it became apparent that analysing 100 generations from an initial no-suicide state generated artifactual temporal clusters as suicides emerged during the first few generations due to social learning, homophily or other processes. Given that real-life suicide cluster data does not start arbitrarily at zero suicides, in the agent-based model 110 generations were run in total, the first 10 generations were ignored and generations 11–110 analysed for clusters.

Each agent is initially given the same fixed probability of committing suicide, *p_0_*. This baseline probability is then modified according to a set of risk factors, intended to capture individual differences in suicide rates. For example, data from the U.S. [31] suggest risk factors of gender (men are 3.9 times more likely to commit suicide than women), ethnicity (white people are 2.2 times more likely than non-white people) and age (over 65s are 1.5 times more likely than 15–24 yr olds). These risk factors appear to combine additively, e.g. white men aged over 65 have the highest compounded risk of suicide. Risk factors are represented in the model as a set of six binary bits, *k_i_*, where *i* indexes the six risk factors (*i* = {1, 2…6}) and *k_i_* ∈ {1−*q*, 1+*q*}. Each bit therefore indicates whether an agent is at higher (1+*q*) or lower (1−*q*) risk of suicide (e.g. male vs. female), and are randomly generated for each agent (except in the case of homophily, see below). The probability of suicide after modification by the risk factors, *p_1_*, is then the product of these risk factors (Equation 1). 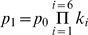
(1)


**Figure 1:**
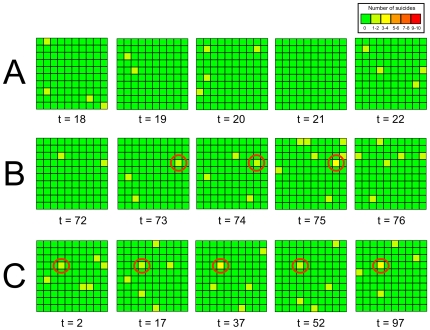
Three time series indicating (A) baseline suicide occurrences with no clustering, (B) a spatiotemporal cluster resulting from social learning, and (C) a spatial cluster resulting from homophily. Each square within the 10×10 grid indicates one 10-agent sub-group, with the colour of the square indicating the frequency of suicide from green (0%) to red (100%). In A, randomly distributed suicide events can be observed due to the non-copycat probability of suicide (*p_0_* = 0.005). No clustering is detected under these conditions. In B, a spatiotemporal point cluster generated by social learning (*s* = 5) is marked with a red circle, and can be seen persisting over a period of three generations from t = 73 to t = 75 inclusive, thus showing localisation in both time and space. In C there is no social learning (*s* = 0), but homophily (h = 1) and large inter-group differences (q = 0.4) causes one sub-group, marked with a red circle, to be composed entirely of high suicide risk agents. This group repeatedly features suicides throughout the simulation run, forming a spatial (but not temporal) cluster despite the lack of social learning.

The magnitude of *q* thus determines the individual variation in *p_1_* within the population. Except where indicated otherwise, in the simulations below *q* = 0.2; six risk factors with *q* = 0.2 gave a suitable range of individual variation across the population, from *p_0_* (1−*q*)^6^ = 0.26*p_0_* to *p_0_* (1+*q*)^6^ = 2.99*p_0_*. Obviously risk factors in the real world are much more complex than this (e.g. age is continuous not dichotomous and there may be more or less than six factors that may interact non-independently). However, the above implementation captures the essential phenomena of individual differences in risk factors in an abstract, simplified way that is easily implemented *in silico*. An example time series with a small baseline risk of suicide of *p_0_* = 0.005 and individual differences of *q* = 0.2 is provided in Figure 1A, which shows rare suicide events distributed randomly in time and space.

### Social learning (s)

Whenever an agent commits suicide, it increases the probability that every other agent in its 10-agent group will commit suicide during the following generation according to the parameter *s* (*s*≥0) as in Equation 2, where *p_2_* is the modified probability of suicide following social learning and *x_n_* is the number of agents in the same group in the previous generation who committed suicide.
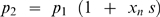
(2)


Thus social learning is assumed to be additive, with each suicide “observed” by an agent increasing their suicide risk *p_2_* in the next generation by an equal amount. Note that this does not apply to the new agent that replaced the suicide agent, or any other new agents in that generation who did not “observe” the suicide in the previous generation. Also note that the (1+ *x_n_ s*) term in Equation 2 constitutes a measure of relative risk, RR (where RR  =  *p_2_*/*p_1_*): when there is no social learning (*s*  =  0) then there is a relative risk of 1, and exposed individuals have the same probability of suicide as unexposed individuals; the relative risk then increases as *s* increases.

Social learning within local groups is predicted to result in the reliable spatiotemporal clustering of suicides as agents acquire suicide behaviour from members of their local group. Table 2 shows the incidence of spatial, temporal and spatiotemporal suicide clusters under different values of both *p_0_* and *s*. First, note that clusters never occur when *s* = 0, i.e. in the absence of social learning (illustrated in Figure 1A). As *s* increases in magnitude, the probability of observing clusters increases, but only for sufficiently large values of *p_0_* (*p_0_* = 0.005 or *p_0_* = 0.01). For these values, spatiotemporal clusters are most likely to emerge, followed by purely spatial clusters, and then purely temporal clusters (e.g. for *p_0_* = 0.005 and *s* = 5: X_s_ = 0.2; X_t_ = 0; X_st_ = 0.8). Figure 1B shows a time series of an example spatiotemporal point cluster, in which a single group temporarily exhibits disproportionately more suicides than surrounding groups. These results therefore support Hypothesis 1a that social learning generates suicide clusters, and specifically spatiotemporal point clusters.

**Table 2:** Suicide clustering in response to varying the baseline (non-copycat) suicide risk (*p_0_*) and the strength of social learning (*s*).

p_0_	s	X_s_	X_t_	X_st_
**0.001**	**0**	0	0	0
	**1**	0	0	0
	**2**	0	0	0.1
	**3**	0	0	0
	**4**	0	0	0
	**5**	0	0	0.1
**0.005**	**0**	0	0	0
	**1**	0	0	0
	**2**	0	0	0.2
	**3**	0.1	0	0
	**4**	0.4	0	0.5
	**5**	0.2	0	0.8
**0.01**	**0**	0	0	0
	**1**	0	0	0
	**2**	0	0.1	0.3
	**3**	0.1	0.1	0.9
	**4**	0.5	0.5	1
	**5**	1	0.4	1

Parameters are the baseline (non-copycat) suicide rate (*p_0_*), the strength of social learning (*s*), and the frequency of spatial (X_s_), temporal (X_t_) and spatiotemporal (X_st_) clusters in replicate simulation runs ranging from 0 (no clustering) to 1 (maximum clustering). Other parameters: *q* = 0.2, *c_p_* = 0, *c_s_* = 0, *h* = 0, *m* = 0, *r* = 0.

### Homophily (h)

To simulate homophily, new agents created at the beginning of the simulation copy the *k_i_* bits of a previously created agent in the same group with a probability *h* (0≤*h*≤1). The first agent in the group takes random *k_i_* values as described above. Thus where *h* = 0 there is no homophily and agents never share bits beyond that expected by chance. Where *h* = 1 there is strict homophily: every agent in the same group shares identical *k_i_* bits and different groups vary in their bits (i.e. no within-group variation and high between-group variation). As some of these groups will by chance have uniformly high risk factors due to the variation caused by *q*, these are the groups we would expect to form suicide clusters even with no social learning. New agents introduced to replace agents that have committed suicide take the same *k_i_* bits of a randomly selected agent in their group in order to maintain the same level of homophily throughout the simulation run. The use of binary bits to simulate homophily is based in part on previous agent-based simulations [32], [33], although in the present model homophily is assumed to have occurred before the simulations begin, rather than emerging during the simulations.

Table 3 shows the probability of observing clusters in response to different levels of *h* and the parameter *q* (the extent of individual differences in baseline suicide risk), which was found to strongly moderate the effect of *h*. When there is zero individual variation in suicide risk (*q* = 0) then no clusters are observed even under maximum homophily (*h* = 1). As *q* increases, clustering becomes more frequent under high levels of homophily. Here, purely spatial clusters are more common than spatiotemporal clusters, while purely temporal clusters are never observed (e.g. for *q* = 0.2 and *h* = 0.75: X_s_ = 0.7; X_t_ = 0; X_st_ = 0.2). An example of a homophily-generated spatial cluster is illustrated in the time series in Figure 1C, in which a single high-risk group repeatedly experiences a disproportionately high frequency of suicides throughout the entire simulation run. The model therefore lends only partial support to Hypothesis 1b, that homophily on suicide risk factors can mimic the spatiotemporal clustering shown above to result from social learning, with the two qualifications that (i) individual differences in suicide risk factors must be sufficiently large and (ii) while spatiotemporal clusters are observed, purely spatial clusters are more likely to be observed, the reverse of that documented for social learning, in which spatiotemporal clusters are more likely than purely spatial clusters.

**Table 3:** Suicide clustering in response to homophily (*h*) in the absence of social learning and under varying levels of individual variation in (non-copycat) suicide risk factors (*q*).

*q*	*h*	X_s_	X_t_	X_st_
**0**	**0**	0	0	0
	**0.25**	0	0	0
	**0.5**	0	0	0
	**0.75**	0	0	0
	**1**	0	0	0
**0.2**	**0**	0	0	0
	**0.25**	0	0	0
	**0.5**	0	0	0
	**0.75**	0.2	0	0.1
	**1**	0.7	0	0.2
**0.4**	**0**	0	0	0
	**0.25**	0	0	0
	**0.5**	0.1	0	0.1
	**0.75**	0.7	0	0.2
	**1**	1	0	1

Parameters are the probability of homophily (*h*), the individual variation in suicide risk (*q*), and the frequency of spatial (X_s_), temporal (X_t_) and spatiotemporal (X_st_) clusters in replicate simulation runs ranging from 0 (no clustering) to 1 (maximum clustering). Other parameters: *p_0_* = 0.005, *s* = 0, *c_p_* = 0, *c_s_* = 0, *m* = 0, *r* = 0.

### Prestige bias (c)

Two parameters were used to simulate a minority of prestigious “celebrities” whose suicides have an increased social influence on other agents' suicide risks. These parameters are *c_p_* (0≤*c_p_*≤1), which specifies the probability that a new agent is assigned celebrity status, and *c_s_* (*c_s_*≥0), which specifies the increase in *p_1_* of another agent in the same group as a result of observing a celebrity agent committing suicide in the previous generation. Thus if *x_n_* is the number of non-celebrity agents in a particular group who in the previous generation committed suicide, and *x_s_* is the number of celebrity agents in the same group who committed suicide in the previous generation, then the suicide risk of surviving agents in that group is now given by Equation 3.
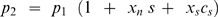
(3)


Thus *c_s_* replaces *s* for celebrity agents, and prestige bias is operating when *c_s_* > *s* such that celebrities have a greater social influence than non-celebrities.

Table 4 shows the effect of prestige bias on the probability of clustering, assuming values of *p_0_* and *s* that would normally not generate clustering (*p_0_* = 0.01, *s* = 1; see Table 2). Increasing the strength of prestige bias *c_s_* increases the probability of observing spatiotemporal clusters, and to a lesser extent purely spatial and purely temporal clusters (e.g. for *c_s_* = 20 and *c_s_* = 0.1: X_s_ = 0.3; X_t_ = 0.2; X_st_ = 1). However, the strength of prestige bias (*c_s_*) must be substantially larger than the non-prestige social learning strength (*s*), with a 20-fold increase in suicide risk in response to celebrity suicides needed to reliably generate spatiotemporal point clusters. Moreover, Table 4 also shows that the strength of prestige bias must be larger as the proportion of agents who are prestigious celebrities gets smaller (i.e. *c_p_* decreases). Overall, then, prestige bias can mimic non-prestige biased social learning in generating spatiotemporal clusters when prestige bias is sufficiently strong to counteract the lower frequency of prestige-based suicides. Hypothesis 2a, however, states that prestige bias alone should generate mass (temporal) clusters rather than spatiotemporal clusters, and thus was not supported by the model.

**Table 4:** Suicide clustering in response to different proportions (*c_p_*) and strengths (*c_s_*) of prestige bias.

*c_p_*	*c_s_*	X_s_	X_t_	X_st_
**0.01**	**5**	0	0	0
	**10**	0	0	0.2
	**20**	0.1	0	0.2
**0.05**	**5**	0	0	0.1
	**10**	0.1	0	0.1
	**20**	0.2	0.2	0.7
**0.1**	**5**	0	0.1	0.3
	**10**	0.2	0.1	0.5
	**20**	0.3	0.2	1

Parameters are *c_p_*, the probability that an agent is a prestigious ‘celebrity’, *c_s_*, the strength of social influence for celebrities, and the frequency of spatial (X_s_), temporal (X_t_) and spatiotemporal (X_st_) clusters in replicate simulation runs ranging from 0 (no clustering) to 1 (maximum clustering). Other parameters: *p_0_* = 0.01, *s* = 1, *q* = 0.2, *h* = 0, *m* = 0, *r* = 0.

### Similarity bias (m)

Here it is assumed that agents only influence each others' probability of suicide if they share at least *m* (0≤*m*≤6) of the six *k_i_* bits that describe individual differences in risk factors. When *m* = 0, none of the *k_i_* bits need to be shared, and similarity bias is not operating. When *m* = 6, learners and models must share all six *k_i_* bits in order for the learner's *p_2_* to be affected by *s*. Thus the higher the value of *m*, the stronger is the similarity bias (i.e. the more similar the model must be to the learner in order for the learner to be influenced by their behaviour).

Table 5 shows that increasing *m* from 0 (no similarity bias) to 6 (agents must be identical to engage in social learning) reduces the frequency of all types of clusters, with no clusters occurring in the extreme case where *m* = 6 (X_s_ = X_t_ = X_st_ = 0). This might be expected, given that similarity bias reduces the set of models from whom suicide behaviour can be learned. Given that social learning generates clusters (Table 2), in blocking social learning similarity bias also eliminates clusters. Hypothesis 2b, that similarity bias generates temporal (mass) suicide clusters, is therefore not supported. However, Table 5 also shows that homophily (*h* = 1) removes the inhibitory effect of similarity bias, with clusters virtually universally observed for large values of *m* (e.g. for *h* = 1 and *m* = 6: X_s_ = 1; X_t_ = 0.8; X_st_ = 1). This is to be expected: when *h* = 1, all agents within a sub-group are identical, and so even when similarity bias is at its strongest (*m* = 6) social learning still occurs. However, given that these clusters are spatial as well as temporal, and that homophily acts to partially mask the clustering effect of social learning, this further undermines Hypothesis 2b that similarity bias generates mass (temporal only) clusters as a result of social learning.

**Table 5:** Suicide clustering in response to similarity bias (*m*) in the absence of homophily (*h* = 0) and when homophily is operating (*h* = 1).

*h*	*m*	X_s_	X_t_	X_st_
**0**	**0**	1	0.4	1
	**2**	0.4	0.4	1
	**4**	0.1	0.3	0.3
	**6**	0	0	0
**1**	**0**	1	0.7	1
	**2**	1	0.5	1
	**4**	1	0.8	1
	**6**	1	0.8	1

Parameters are the probability of homophily (*h*), the strength of similarity bias (*m*), and the frequency of spatial (X_s_), temporal (X_t_) and spatiotemporal (X_st_) clusters in replicate simulation runs, ranging from 0 (no clustering) to 1 (maximum clustering). Other parameters: *p_0_* = 0.01; *s* = 5, *q* = 0.2, *c_p_* = 0, *c_s_* = 0, *r* = 0.

### One-to-many transmission (r)

The one-to-many transmission consequences of the mass media is simulated by manipulating the radius of a “zone of social influence” across which social learning of suicide behaviour occurs. Thus when an agent commits suicide, in the following generation every agent in every group that is within *r* (0≤*r*≤9) sectors from the suicide agent's group has their suicide probability *p_1_* updated according to Equation 3. Where *r* = 0, only the suicide agent's group is affected, as assumed in all of the simulations discussed previously. Where *r* = 1, every agent in the eight groups immediately surrounding the suicide agent's group is affected (or fewer groups if the focal group is on the edge of the grid). In the extreme case where *r* = 9, the zone of social influence encompasses the entire grid and all 1000 agents in the population are affected by every suicide.

**Figure 2:**
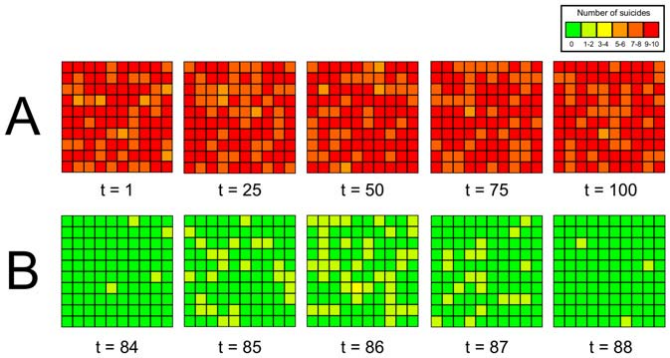
Two time series illustrating the effects of strong one-to-many transmission (*r* = 9). In A, when the baseline suicide rate and the strength of social learning are relatively high (*p_0_* = 0.005, s = 1), a pandemic causes the entire population to commit suicide at extremely high rates throughout the simulation run. Neither spatial nor temporal clusters are observed under these conditions, which are obviously highly unrealistic. In B, when the frequency of social learning is reduced by introducing prestige bias (*p_0_* = 0.005, *s* = 0, *c_p_* = 0.01, *c_s_* = 5) such that only a small minority of agents have social influence, mass (temporal but not spatial) clusters emerge. Here, one of the four suicides that occur in generation t = 84 was a prestigious “celebrity”, resulting in a mass cluster in the following three generations. Suicide rates then drop back to baseline pre-cluster levels at generation t = 88.

Table 6 shows that, for values of *p_0_* and *s* that would not normally produce clusters (*p_0_* = 0.005, *s* = 1), a small increase in *r* increases the probability of detecting clusters, predominantly spatial and spatiotemporal clusters (e.g. for *r* = 3: X_s_ = 1; X_t_ = 0.6; X_st_ = 1). This is because there are now more agents who are affected by *s*, thus increasing the probability of a cluster occurring. However, large values of *r* fail to generate clusters of any kind (e.g. for r = 9: X_s_ = X_t_ = X_st_ = 0). The reason clusters were not observed at large values of *r* was that the widespread social learning causes a suicide pandemic such that virtually the entire population constantly committed suicide during every generation. Such a pandemic is illustrated in Figure 2A. As suicide rates are at a constantly high rate, there are no clusters in either time or space. Obviously, such a pattern of constant mass suicide is highly unrealistic. Overall, Hypothesis 2c, that one-to-many transmission generates mass clusters, was therefore not supported under any of these values of *r*.

**Table 6:** Suicide clustering in response to one-to-many transmission (*r*).

*s*	*M*	*c_p_*	*c_s_*	*r*	X_s_	X_t_	X_st_
**1**	**0**	**0**	**0**	**1**	0.4	0.2	0.5
				**3**	1	0.6	1
				**6**	1	0	1
				**9**	0	0	0
**0.1**	**0**	**0**	**0**	**1**	0	0	0
				**3**	0.1	0	0
				**6**	0.1	0.4	0.1
				**9**	0	0.7	0.3
**1**	**5**	**0**	**0**	**1**	0	0	0
				**3**	0	0	0
				**6**	0	0.6	0.1
				**9**	0	0.8	0.3
**0**	**0**	**0.01**	**5**	**1**	0	0	0
				**3**	0	0.3	0.4
				**6**	0.1	1	1
				**9**	0	1	0.9

Parameters are the strength of social learning (*s*), the range of one-to-many transmission (*r*), the strength of similarity bias (*m*), the proportion of prestigious celebrities (*c_p_*), the strength of prestige bias (*c_s_*), and the frequency of spatial (X_s_), temporal (X_t_) and spatiotemporal (X_st_) clusters in replicate simulation runs, ranging from 0 (no clustering) to 1 (maximum clustering). Other parameters: *p_0_* = 0.005, *h* = 0, *q* = 0.2.

However, Table 6 also shows three cases where mass clusters *were* observed. In these cases the effect or frequency of copycat suicide is reduced such that suicide pandemics fail to take off, yet copycat suicides are not so weak or infrequent that clusters do not occur. The first is when the strength of social learning (*s*) is directly reduced (e.g. for *r* = 9 and *s* = 0.1: X_s_ = 0; X_t_ = 0.7; X_st_ = 0.3). The second is where similarity bias operates to reduce the frequency of social learning events (e.g. for *r* = 9, *m* = 5: X_s_ = 0; X_t_ = 0.8; X_st_ = 0.3). The third is where prestige bias reduces the subset of agents who have social influence (e.g. for *r* = 9, *c_p_* = 0.01, *c_s_* = 5: X_s_ = 0; X_t_ = 1; X_st_ = 0.9). In each of these cases the probability of pandemics such as those observed in Figure 2A is reduced either by reducing the strength of social learning (*s* = 0.1) or reducing the frequency of social learning events (*c_p_* = 0.01 or *m* = 5). Instead, temporary clusters occur that are localised in time before returning to baseline suicide rates. When *r* is large, these clusters affect all agents in the population equally and so are not spatially localised. Such a mass cluster is illustrated in Figure 2B. Hypothesis 2c is therefore supported only under the conditions where one-to-many transmission is strong enough to eliminate spatial clustering *and* where social influence is strong enough to generate statistically significant clusters yet not so strong as to cause population-wide suicide pandemics.

## Discussion

Evidence accumulated during recent years suggests that suicide may be subject to social learning, potentially resulting in distinct clustering of suicides in time and/or space. Point clusters are clusters of suicides in both time and space, and have been attributed to social learning within local groups [5]. Mass clusters are clusters of suicides in time but not space, and have been attributed to prestige and similarity bias (preferentially copying prestigious or similar models: [19]) and the mass media [11], [23]. The present study used agent-based modelling techniques, in combination with rigorous statistical cluster-detection analyses, to assess the validity of these proposals. Naturally, abstract simulation models cannot give definitive answers to questions concerning copycat suicides that are ultimately empirical. However, they can help to clarify definitions of different processes with greater precision than informal verbal explanations, they can lend plausibility to hypotheses by demonstrating that assumed consequences logically follow from premises, and they can guide future empirical work by identifying the kinds of variables that might be important and that future empirical work should focus on.

The prediction that social learning within groups of agents generates spatiotemporal point clusters was supported. An additional hypothesis, that homophily generates spatiotemporal clusters in the absence of social learning because individuals who are independently at high risk of suicide congregate in space and form non-social suicide clusters [3], was only partially supported. Homophily only generated clusters when there was relatively high individual variation in agents' (non-copycat) suicide risk, such that high-risk clusters occur. Furthermore, these homophily clusters were most likely to be spatial, to a lesser extent spatiotemporal, and never purely temporal. This makes sense given that groups maintained their relative levels of risk throughout the simulation, and there is no reason why the agents would cluster their suicides in time without social learning. These findings might be used to guide future empirical tests of Joiner's [3] homophily hypothesis, by specifically taking into account the degree of individual variability in known suicide risk factors (e.g. age, sex, ethnicity) in a region, and by distinguishing between the spatial-but-not-temporal clusters generated by homophily and the spatiotemporal clusters generated by social learning.

A second set of simulations found that neither prestige bias (preferentially copying prestigious celebrities) nor similarity bias (preferentially copying others who are similar to oneself) generate mass (temporal-but-not-spatial) clusters alone. Both prestige and similarity bias act to reduce the subset of potential models from whom suicide-related behaviour can be learned. For prestige bias, this is because only a minority of the population can be, by definition, prestigious. For similarity bias, requiring that models must be similar to oneself in some respect reduces the number of potential models from whom one can learn. Both biases therefore reduce the frequency of social learning events and reduce the probability of clustering. This reduction in the probability of clustering was counteracted under certain conditions, such as increasing the strength of prestige bias and introducing homophily, which made neighbouring agents similar to one another and therefore more likely to copy each other even at high levels of similarity bias. Yet even under these conditions (strong prestige bias, homophily) mass clusters were no more likely to emerge than purely spatial clusters or spatiotemporal clusters.

However, the mass media, represented here by one-to-many transmission, *did* generate mass clusters, but only under certain conditions. When social influence was too strong, extensive one-to-many transmission gave rise to suicide pandemics in which all agents committed suicide with an extremely high probability. These pandemics neither contained any clusters nor were very realistic. Mass clusters did emerge, however, when social influence was weak, either directly via a reduced strength of social learning, or indirectly via prestige bias or similarity bias, which both reduced the subset of models that agents could be influenced by. In summary, prestige and similarity bias were neither necessary nor sufficient for mass clusters, while one-to-many transmission was necessary but not sufficient. The three processes in combination generated mass clusters, which is consistent with sociological evidence for each in actual cases of mass suicide clusters. However, the model highlights the very different roles that each plays: one-to-many transmission acts to spread suicide behaviour across the entire population thus eliminating spatial clustering, while prestige and similarity bias somewhat counter-intuitively (and in contrast to previous suggestions: [19]) prevent copycat suicides from persisting and becoming pandemic.

Obviously several assumptions of this model are extreme simplifications of a complex real-life phenomenon. For example, the implementation of prestige and similarity bias in the present model only incorporated certain, simplified aspects of these processes, ignoring for example potential runaway prestige effects [22], prestige hierarchies [20] and the consequences of similarity bias on individual variation [32], [33]. There is also no consideration of the mechanism by which ‘social influence’ occurs: social influence via the transmission of practical knowledge regarding suicide methods might have quite different consequences to social influence via the emotional effect of a close friend's suicide. A further source of potential inaccuracy is the mismatch between parameter values in the model and equivalent real-life estimates. The baseline suicide rate that is required in the model (0.001≤*p_0_*≤0.01) to detect statistically significant clusters is higher than actual national suicide rates (e.g. 11 in 100,000, or 0.00011 in the USA in 2005: [31]), although this is possibly because of the much smaller population size in the model compared to actual national populations. The assumed strength of social influence *s* might also be considered large (e.g. *s* = 5, or for prestige bias *c_s_* = 20) compared to estimates that publicised suicide stories increase the national suicide rate by just 2.5% [10] or that only 2–4% of suicides show any spatiotemporal clustering [5]. However, it should be noted that under some conditions of the model much smaller values of *s* reliably generated clustering (e.g. when *r* = 9, clusters occurred when *s* = 0.1), and more detailed individual-level studies have found relatively large estimates of social influence. For example, one study found that teenagers who knew another person who had committed suicide were three times more likely to commit suicide than teenagers who did not know anyone who had committed suicide [34]. However, even with simplified assumptions and exaggerated parameter values, the findings reported above can be useful in showing qualitatively how a change in one variable (e.g. the magnitude of individual differences) interacts with another (e.g. homophily) to cause some effect (e.g. increased spatial clustering). These relationships can then be tested in actual datasets.

In supporting the assumptions made by sociologists that point and mass clusters can be taken as evidence that suicide may spread via social learning, the model reinforces the need for efforts to counter the social transmission of suicide-related information. The findings related to point clusters suggests that social learning and homophily generate distinct types of clusters (predominantly spatiotemporal versus predominantly spatial); by using this knowledge to distinguish between copycat point clusters and homophilous point clusters, efforts to reduce social transmission might be more effectively targeted at the former. The findings related to mass clusters in particular highlight the need for media guidelines that restrict the dissemination and glorification of suicides, as already introduced in many countries [11], [23]. More specifically, the model suggests that increasing the range of one-to-many transmission (*r*), increasing the social influence of prestigious celebrities (*c_s_*) and increasing the proportion of the population who are assigned celebrity status (*c_p_*) can all increase the probability of widespread suicide pandemics. Anecdotally, all three of these trends appear to be occurring in many countries in recent years: satellite television and the internet have increased the global range of the mass media; celebrities such as film actors and pop singers are being assigned increasing importance relative to politicians and intellectuals (whose suicides do not elicit copycat suicide attempts); and reality television programmes are increasing the number of celebrities within society. This highlights how media guidelines on suicide reporting will become all the more important in the future.

## Supporting Information

Supplementary File S1 The agent-based model (as a zipped.exe file) used to generate the results in the paper.
(0.35 MB ZIP)
